# Age‐related changes in the gut microbiota of the Chinese giant salamander (*Andrias davidianus*)

**DOI:** 10.1002/mbo3.778

**Published:** 2018-12-25

**Authors:** Mengjie Zhang, Sarah Gaughan, Qing Chang, Hua Chen, Guoqing Lu, Xungang Wang, Liangliang Xu, Lifeng Zhu, Jianping Jiang

**Affiliations:** ^1^ Chengdu Institute of Biology Chinese Academy of Sciences Chengdu China; ^2^ College of Life Sciences Nanjing Normal University Nanjing China; ^3^ Department of Biology University of Nebraska at Omaha Omaha Nebraska; ^4^ Shanghai Biozeron Bioinformatics Center Shanghai China

**Keywords:** age, Chinese giant salamander, gastrointestinal tract, microbial community changes, nutritional source shift

## Abstract

The composition of the intestinal microbial community may vary across developmental stages. In this study, we explored how this microbial community shifted along the intestinal tract of the Chinese giant salamander (*Andrias davidianus*) at various ages. Next‐generation sequencing was used to sequence the bacterial 16S rRNA gene from different kind of samples, including the stomach, duodenum, ileum, and rectum. The highest mean relative abundance of the bacterial community in the gastrointestinal tract shifted in relation to age: within the first year, Bacteroidetes (47.76%) dominated the gut microbiome, whereas Proteobacteria was the most dominant at age 2 (32.88%) and age 3 (30.78%), and finally, Firmicutes was the most dominant at age 4 (34.70%). The overall richness of the gut bacterial community also generally increased from age 2 to 4. Hierarchical cluster analysis revealed that the gut microbiome at age 2 had greater variability than that at either age 3 or 4, likely representing a shift in diet from yolk or redworms as a juvenile to shrimp or crab as an adult. As these salamanders develop, their gastrointestinal tracts increase in complexity, and this compartmentalization may also facilitate an increase in microbial gut diversity.

## 1 INTRODUCTION

There are trillions of microorganisms living within multicellular organisms’ gastrointestinal (GI) tracts. These microbial communities play essential roles in the metabolism, physiology, ecology, and even evolution of their hosts (Colston, 2017; Colston, Noonan, & Jackson, 2015; Kohl & Carey, 2016; Zhu, Wu, Dai, Zhang, & Wei, 2011). A large amount of microorganismal research has centered on vertebrates (Ellis & McSweeney, 2016; Ley, Lozupone, Lozupone, Hamady, Knight, & Gordon, 2008); however, amphibians have been neglected and are potential model animals in gut microbial studies (Knutie, Wilkinson, Wilkinson, Kohl, & Rohr, 2017). Amphibians represent a unique group and are currently experiencing severe population declines and extinctions primarily due to habitat destruction, environmental pollution, overexploitation, and emerging disease spread (Jiang et al., 2016). Previous research has focused on mitigating a devastating amphibian fungal pathogen, *Batrachochytrium dendrobatidis*, by focusing on cutaneous bacteria or antimicrobial peptides (Bai, Liu, Fisher, Garner, & Li, 2012; Briggs, Knapp, & Vredenburg, 2010; Colston & Jackson, 2016; Jiménez & Sommer, 2016; Ley, Hamady, et al., 2008).

A detailed understanding of how an organism's gut microbiome community is formed and utilized across an organism's lifespan is essential to understand how anthropogenic and natural disturbances affect imperiled amphibian species. Some of the factors that dictate the composition of an organism's gut microbiome include phylogeny (Vences, Lyra, Kueneman, & Bletz, 2016), dietary preference and prey availability (David et al., 2014; Knutie, Shea, et al., 2017; Ley, Lozupone, et al., 2008; Zhang et al., 2010), endocrine disruptors (Vences et al., 2016), metamorphic transition from the larval stage (tadpole) to the adult (frog) stage in Anura (Kohl, Cary, Karasov, & Dearing, 2013; Vences et al., 2016) and internal regulation facilitating hibernation (Weng, Yang, & Wang, 2016). There are many confounding factors in metamorphosis for amphibians, such as drastic remodeling of the digestive tract, dietary shifts, and changes in the physiological index in the digestive tract. All of these complex changes at different ages or during metamorphosis make it challenging to identify the direct or crucial effects of gut microbiome alterations. The gut microbiota of amphibians may affect the mucosal immunity (Colombo, Scalvenzi, Benlamara, & Pollet, 2015). More concretely, members of the gut microbiota can influence immunity during gastrointestinal development (Rodríguez et al., 2015; Round & Mazmanian, 2009; Wu & Wu, 2012). In addition, other gut microbial symbionts may disproportionately alter the assembly of gut microbiomes through priority effects. For example, early disruption of the gut microbiota in the Cuban tree frog (*Osteopilus septentrionails*) has been demonstrated to decrease the resistance of individual frogs to parasites (Knutie, Shea, et al., 2017). These intrinsic microbiome studies have received considerable attention.

The Chinese giant salamander (*Andrias davidianus*) is a species that has been classified as a class II critically endangered species on the national list of protected animals in China. The Chinese giant salamander is often called a living fossil and is considered a valuable model species for phylogenetic and evolutionary studies (Geng et al. 2017). Giant salamanders are susceptible to bacterial infections (Meng, Zeng, Yang, & Xiao, 2009). Thus, study of intestinal microorganisms in giant salamanders has become extremely urgent.

In this paper, we choose captive Chinese giant salamanders as a representative of Urodela and treat age (development), accompanied by a shift in dietary preferences, as a driving force of the biological evolution of gut microorganisms. We intend to lay a foundation for the conservation biology of giant salamander and provide a baseline for future infectious disease research.

## 2 MATERIALS AND METHODS

### 2.1 Sample collection and gut content preparation

A total of 135 individual Chinese giant salamanders ranging from age 1 to 4 (Appendix 1) were collected from a farm located in Lueyang County in Shanxi Province in December 2016. During their first year of life, Chinese giant salamanders are entirely aquatic and rely solely on the yolk sac for nutrition. After age 2, Chinese giant salamanders continue to depend on the yolk sac for nutrition but begin feeding on redworms supplied by the aquaculture facility. After age 3, they rely solely on external food sources, mainly shrimp and crab.

Individuals aged 1 and 2 were euthanized with MS‐222 at a concentration of 0.6–1.0 g/L for 10–20 min (Wei et al., 2014), and those aged 3 and 4 were euthanized in an enclosed terrarium using 5–10 sterile cotton balls bedewed in ether for approximately 30 min. Following euthanization, body weight and total length were measured (Appendix 1), and then the holonomic gastrointestinal tract was removed from the abdominal cavity and sectioned according to the anatomical compartment when possible, including the stomach, duodenum, ileum, and rectum (Li, Zhang, Ma, & Wang, 1991; Peng, Chen, & Feng, 1998). Dissection tools were changed strictly between individuals and intestinal sections. The contents of each section were immediately gently squeezed into a 2 ml sterile centrifuge tube and then stored at −80°C for DNA extraction. Overall, we obtained 53 gastrointestinal samples (Appendix 2).

### 2.2 DNA extraction and bacterial 16S rRNA sequencing

Gastrointestinal samples were thawed on ice, and microbial genomic DNA was extracted using a QIAamp Fast DNA Stool Mini Kit (QIAGEN, Hilden, Germany) according to the manufacturer's protocol. The integrity of DNA was visually assessed using 1.0% agarose gel electrophoresis and quantified using a Qubit and NanoDrop. The highly variable V4 region of the 16S rRNA gene was amplified from community genomic DNA using the bacterial‐specific universal primers 515F (GTGCCAGCMGCCGCGGTAA) and 806R (GGACTACHVGGGTWTCTAAT). PCR was performed in triplicate using a 25 μl reaction containing 2 μl DNA template, 2.5 μl 10× TransStart Taq buffer, 1 μl each of forward and reverse primers, 2 μl dNTPs (2.5 mM), 0.25 μl TransStart Taq DNA Polymerase, and 16.25 μl ddH_2_O. The PCR amplification conditions were as follows: initial denaturation at 94°C for 5 min, followed by 35 cycles of denaturation at 94°C for 30 s, annealing at 53°C for 30 s and elongation at 72°C for 30 s, and finally, a final extension at 72°C for 10 min. PCR products were purified with a Universal DNA Purification Kit (TIANGEN), and barcoded V4 amplicons were sequenced using the Illumina HiSeq platform (HiSeq2500 PE250).

### 2.3 Raw data processing and statistical analysis

Raw sequences were generated from the Illumina HiSeq sequencing platform. We performed quality control (e.g., demultiplex and denoise) and taxon classification in QIIME2 (https://docs.qiime2.org/2018.8/). Finally, we obtained OTU (operational taxonomic unit) abundance tables and diversity results for downstream analysis. We chose to rarefy our sampling depth at ~42,000 to equalize the sampling depth across all samples. The significant taxa and alpha diversity among ages or sections were analyzed using one‐way analysis of variance (ANOVA) in SPSS Statistics 20.0 (SPSS, 2011) and Stamp 2.1.3 (Parks, Tyson, Hugenholtz, & Beiko, 2014). The differences in body weight and total length of individuals were analyzed using the Kruskal–Wallis test. The variation in the microbial composition (genera abundance) among groups was used to generate NDMS (nonmetric multidimensional scaling) in PAST3 (Hammer, Harper, & Ryan, 2001). The heatplus package (Ploner, 2012) in R was used to generate a Heatmap for the predominant genera in these 53 samples.

Moreover, to evaluate the effect of either intestinal section or age across these 53 samples, we performed one‐way PERMANOVA on Bray‐Curtis dissimilarities in PAST3 (Hammer et al., 2001) to test the microbial community composition. Because there was only one sample from age 1 (pooled individuals), the analysis did not include this sample.

## 3 RESULTS

The sequencing reads of the bacterial 16S rRNA gene resulted in 3,443,705 qualified sequences from 53 gastrointestinal samples. We chose to rarefy our sampling depth at ~42,000 to equalize the sampling depth across all samples (Appendix 3). These high‐quality sequences clustered into an average of 1,611 OTUs based on the 97% sequence similarity. We identified 61 phyla, 681 families, and 1,810 genera from these OTUs (Appendix 4).

### 3.1 Alpha‐diversity of the intestinal microbiota with age

**Figure 1:**
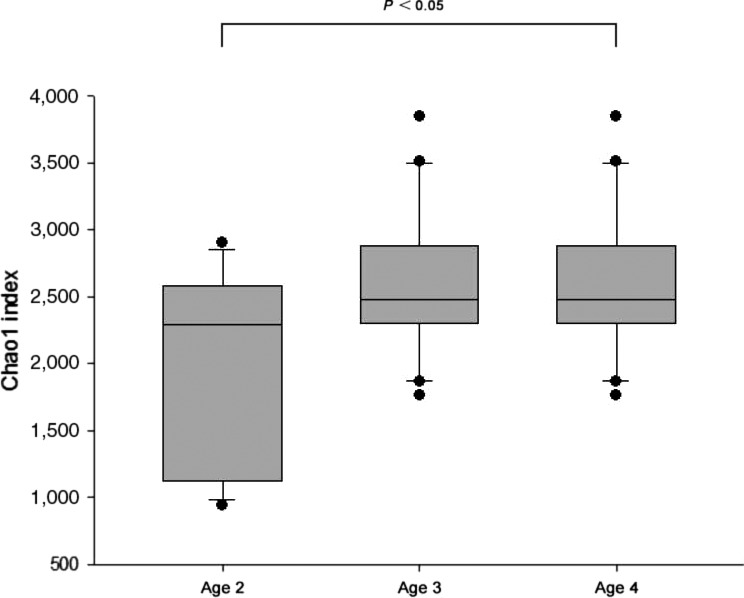
Chao 1 index of samples from gastrointestinal samples from ages 2 to 4 individuals

The Shannon, Chao 1 and Ace indexes were calculated for each of the 53 gastrointestinal samples. The diversity and richness index in gastrointestinal samples tended to increase from age 1 to 4, and minimum and climax diversities were almost always observed in samples from age 2 and 4 individuals, respectively (Table 1). The difference observed in the Chao 1 index of gastrointestinal samples from age 2 to 4 individuals was statistically significant (Turkey HSD, *p* < 0.05), and samples of age 2 individuals had the lowest index (Figure 1).

**Table 1:** Average number (±*SD*) of observed OTUs and the Shannon, Chao 1 and Ace indexes among gastrointestinal samples from age 1 to 4 individuals

Diversity indices	Age 1	Age 2	Age 3	Age 4
Observed OTUs	1,346	1,322 ± 437	1,655 ± 471	1,819 ± 499
Shannon	3	4 ± 0.57	4 ± 0.87	5 ± 0.84
Chao 1	2,154	2,001 ± 773	2,482 ± 639	2,624 ± 553
Ace	2,662	2,343 ± 980	2,848 ± 785	2,989 ± 604

Note

There are no *SD* values for age 1 due to the shortage of multiple animal samples and lack of gut contents for multiple samples for further sequencing.

### 3.2 Gastrointestinal tract bacterial beta‐diversity

**Figure 2:**
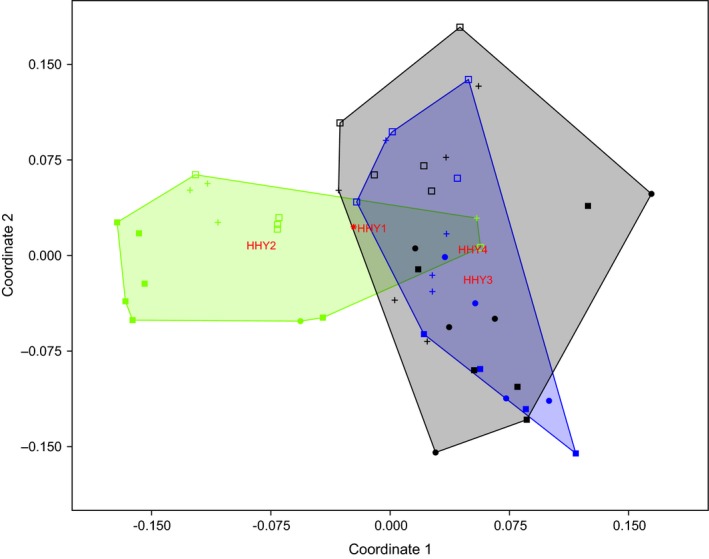
Non‐Metric Multi‐Dimensional Scaling (NDMS) of the dissimilarity (Bray‐Curtis distance on microbial species abundance) in these 53 samples from ages 1 to 4 individuals, including various sections of the gastrointestinal tract (stomach: filled square; duodenum: dot; ileum: plus; rectum: square). Age 1: red and asterisk; age 2: green; age 3: blue; age 4: black. Closure was generated by the convex hull method (Barber, Dobkin, & Huhdanpaa, 1996)

**Figure 3:**
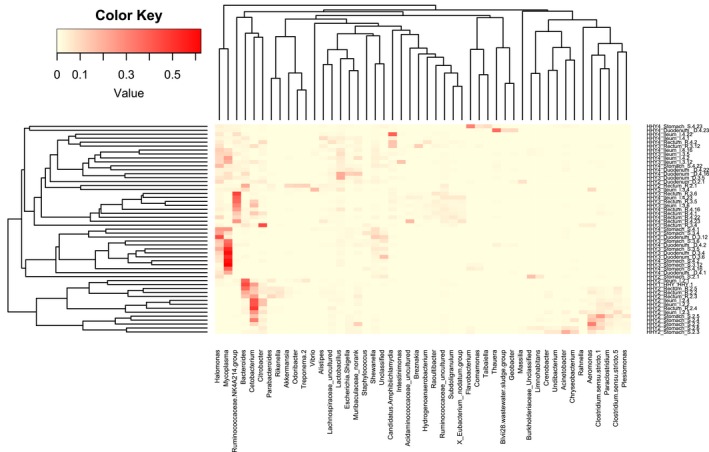
Heatmap of gastrointestinal samples (removing genera with less than 5% as their maximum relative abundance) based on information at the genera level. Columns represent the bacterial genera, and rows represent the 53 gastrointestinal samples. The values (color key) in the heatmap represent the relative abundance of each genus. The tree (left): hierarchical cluster tree assembled according to the Bray‐Curtis distance of the relative abundance of all microbial genera of each sample. The tree (top): hierarchical cluster tree assembled according to the Bray‐Curtis distance of the relative abundance of each genus in these 53 samples

A Bray‐Curtis‐based nonmetric multidimensional scaling (NDMS) plot of gastrointestinal samples revealed a separation between age 2 samples and age 3 and 4 samples (Figure 2). Hierarchically clustered analysis confirmed the alpha‐diversity analysis results that showed that the gastrointestinal bacterial communities of age 2 individuals were unique from those of age 3 or 4 individuals (Figure 3). Cluster tree analysis indicated that stomach samples tended to cluster together (Figure 3 and Appendix 5). The UniFrac‐unweighted distance of the stomach versus duodenum, stomach versus ileum, and stomach versus rectum groups were relatively large compared to that of the groups between other sections except for the stomach (Appendix 6). One‐way PERMANOVA showed a significant difference in microbial composition among intestinal sections (*F* = 2.998, *p* = 0.0003, Appendix 7).

### 3.3 Changes of microorganisms with age

The dominant gastrointestinal microbiota composition of all the sections varied with age (Appendix 8). The top two most prevalent phyla in age 1 samples were Bacteroidetes (47.76%) and Fusobacteria (24.03%), whereas the two most abundant bacterial phyla from age 2 to age 4 samples were Proteobacteria (age 2: 32.88%; age 3: 30.78%; age 4: 27.17%) and Firmicutes (age 2: 22.65%; age 3: 28.90%; age 4: 34.70%). From age 2 to 4, the relative abundance of Actinobacteria, Tenericutes and Chlamydiae significantly increased (Kruskal–Wallis, *p* < 0.05; Appendix 9a, b and f). Bacteroidetes, Verrucomicrobioa and Fusobacteria also showed significant differences and decreased trends (Appendix 9c, d and e). Firmicutes increased from 22.65% to 34.70% between ages 2 and 4. However, this increase was not statistically significant. At the genus level, *Mycoplasma* (0.05%) and *Halomonas *(0.20%) were relatively scarce in age 2 individuals. However, these two genera were the top microbial genera present at ages 3 and 4 (Appendix 10). *Cetobacterium *(2.75%) and *Bacteroides *(1.42%) were prominent at age 2 but relatively rare by ages 3 and 4 (Appendix 10). One‐way PERMANOVA revealed that most of the significant differences were detected between age 2 and other age samples (Appendix 7).

### 3.4 Comparison of the microbial community across gastrointestinal tract sections

The relative abundances of Chlamydiae (Appendix 11a), Fusobacteria (Appendix 11b), and Firmicutes (Appendix 11d) at age 3 across the stomach‐duodenum‐ileum‐rectum were significantly different and tended to increase among these sections. By contrast, the relative abundance of Tenericutes decreased (Appendix 11c). The relative abundances of Proteobacteria (Kruskal–Wallis, *p* < 0.05; Appendix 11f) and Spirochaetes (Kruskal–Wallis, *p* < 0.05; Appendix 11e) at age 4 were significantly different among sections. At age 4, significant differences among various taxa of Aeromonadaceae, Burkholderiaceae, Lachnospiraceae and Mycoplasmataceae were observed between the stomach and other gut chambers combined at the family level (Table 2). Similarly, Ruminococcaceae, Lachnospiraceae and Mycoplasmataceae were significantly different at age 3. The abundances of Bacteroidaceae, Aeromonadaceae, Burkholderiaceae and Mycoplasmataceae were observed among parts at age 2.

**Table 2:** Comparison of the abundant bacterium resident in the stomach and other gut chambers combined at the family level from gastrointestinal samples from age 2 to 4 individuals

Family	Age 2	Age 3	Age 4
Fusobacteriaceae	−	−	−
Bacteroidaceae	+	−	−
Enterobacteriaceae	−	−	−
Aeromonadaceae	+	−	+
Burkholderiaceae	+	−	+
Ruminococcaceae	−	+	−
Lachnospiraceae	−	+	+
Mycoplasmataceae	+	+	+

Note

“+” indicates bacteria whose relative mean abundance between sections are significantly different, and “−” indicates similar taxa.

## 4 DISCUSSION

### 4.1 The shift of the nutritional source with age might be related to the microbiome communities

In this study, we found that the abundance of Firmicutes were increased in age 3 and 4 samples; however, Bacteroidetes were enriched in age 1 and 2 samples. Multiple studies show that a high‐fat diet leads to an increase in Firmicutes and that a high‐fiber diet leads to an increase in Bacteroidetes (Clarke et al., 2012; Turnbaugh et al., 2006). We speculated that these changes in the gut microbiome might be related to the transition between endogenous and exogenous nutrition sources across their development (from age 1–4 years.).

The Fusobacteria content was highest in young Chinese giant salamanders and decreased with age in this study, suggesting that this genus may play a role in the development of young Chinese giant salamander. Previous studies have documented a potential role in protein degradation by Fusobacteria in vertebrates, such as alligators and vultures, that prey primarily on carrion (Colston & Jackson, 2016; Keenan, Engel, & Elsey, 2013; Roggenbuck et al., 2014). The co‐occurrence of Clostridia and Fusobacteria has been documented as allowing their hosts to consume partially decomposed carrion, which often contains toxin‐producing bacteria (Roggenbuck et al., 2014). Some scavenging birds have antibodies against toxins such as botulinum (Ohishi, Sakaguchi, Riemann, Behymer, & Hurvell, 1979). Here, young Chinese giant salamanders (age 2) had a similar pattern in their gut microbiomes: a high abundance of Cetobacterium (belonging to the family Fusobacteria) and *Clostridium *sensu stricto 1 (belonging to the family Clostridiaceae; Figure 3). This gut microbial feature might be associated with their feeding behavior in this study (eating red worms). However, the mechanism of the tolerance of these toxin‐producing bacteria is still unclear.

By the age of 4, we determined that the composition of the microbiomes of Chinese giant salamander primarily shifted from Bacteroidetes bacteria to predominately Firmicutes bacteria. As Chinese giant salamanders age, they switch to shrimp and crabs as their primary food source (ages 3 and 4). A previous study demonstrated that the protein and lipid contents increased with this dietary shift and were highest in samples collected from age 3 and 4 individuals (Liu et al., 2016; Ouyang, Chun, Guangjie, & Jiyong, 2016). A shift in bacterial communities as a result of maturation has been observed in the Leopard frog (*Lithobates pipiens*), in which the non‐acidic stomachs and reduced hind guts in tadpoles shift to acidic stomachs, shorter small intestines and enlarged hind‐gut in adults during metamorphosis (Colston & Jackson, 2016; Hourdry, L'Hermite, & Ferrand, 1996; Kohl et al., 2013). A shift in dietary preference could also account for the changes of microorganisms (Kohl et al., 2013). In our study, the higher diversity and richness of bacteria in age 4 samples may be required to absorb nutrients and increase food intake. In addition, with increasing age, the volumetric increase with a shift in the gastrointestinal microbial community might be a response to the dietary shift and maturation in Chinese giant salamander.

### 4.2 Compartmentalization of the gastrointestinal tract with ages might be related to the microbiome communities

During metamorphosis, the gastrointestinal tract experiences compartmentalization and completely divides into the stomach, duodenum, ileum, and rectum from ages 1 to 4, and each section serves a unique biological function. This compartmentalization, in addition to producing specialized microbial assemblages, may facilitate the extraction of nutrients (Pereira & Berry, 2017). Our study demonstrated that different microbial assemblages are present in each of these subcompartments, which appeared to agree with previous studies in other vertebrates; therefore, these subcompartments contain distinct physiochemical environments that develop diverse microbial assemblages along their total length (Keenan & Elsey, 2015).

### 4.3 Intestinal microorganism dissimilarity across sections

The diversity of bacteria living in the stomach was relatively limited, primarily to Proteobacteria and Tenericutes. In many vertebrates, the stomach mostly plays a role in initially mechanically and chemically breaking down food. Mycoplasma is unable to perform many metabolic functions and are thought to be primarily obligate commensals or parasites (Dandekar et al., 2002). Different *Mycoplasma ribotypes* may dominate in the foregut versus the hindgut, suggesting partitioning by location in the digestive tract of the long‐jawed mudsucker (*Gillichthys mirabilis*; Bano, deRae, Bennett, Vasquez, & Hollibaugh, 2007). The specializations in the gut microflora of silver drummers (*Kyphosus sydneyanus*) may also be tied to feeding (Moran, Turner, & Clements, 2005). Mycoplasma stains from humans grew best in agar from pH 5.5 to 6.5 (Shepard & Lunceford, 1965). Mycoplasma is very host‐ and tissue‐specific, so the high abundance of Mycoplasma and the lowest Shannon diversity in giant salamander stomach content samples may be supported by habitat specialization in the digest system (e.g., the acidic environment of stomach) and reflected the putatively low metabolic functions of stomach symbiotic microbiomes.

Within the posterior gastrointestinal tract, the ileum and rectum harbored more complex microbial assemblages (e.g., high alpha diversity). Previous studies have demonstrated that the neutral pH maintained within this region of the digestive tract offers a more conducive internal environment for the maintenance of larger microbial assemblages than those found in highly acidic stomachs (Lu et al., 2014). The length of the gastrointestinal tract chambers increases significantly following this compartmentalization process. The volumetric increase in food retention time facilitates the digestion of more complex diets (Colombo et al., 2015). In addition to the increase in volume, there is a noticeable increase in the surface area of these chambers and folded mucosa. These large surface areas provide strata for bacterial colonization and the development of biofilms (Keenan & Elsey, 2015).

## 5 CONCLUSION

Our research utilized 16S rRNA gene‐targeted sequencing to demonstrate that microbial assemblages shift as Chinese giant salamander age. Metamorphosis facilitates subcompartmentalization of the digestive tract of Chinese giant salamanders. Metamorphosis is likely a driving force of specialization within the digestive tract, the shift in dietary preferences and the specialization of microbial assemblages within the gastrointestinal tract to maximize nutrient extraction from their new diets. This study was unable to provide a fine scale resolution as to when this shift occurs, particularly between ages 1 and 2. To precisely determine when these shifts occur, future studies should consider the digestive status of each digestive tract environment from more individuals at smaller age intervals**.**

## CONFLICT OF INTEREST

The authors declare that there is no conflict of interest.

## AUTHORS CONTRIBUTION

ZL, JJ, and CQ conceived the project. ZM performed the experiments. ZM, ZL, and HC analyzed the data. ZM, GS, CQ, ZL, and JJ wrote the manuscript. All of the authors gave final approval for publication.

## ETHICS STATEMENT

The animal use protocol in this study (permit: CIBACUC20160305) was reviewed and approved by the Animal Ethical and Welfare Committee of Chengdu Institute of Biology, Chinese Academy of Sciences, China. Chengdu, 610,041, China. The Chairman of this committee is Dr. Xinquan Zhao.

## Data Availability

The sequencing data have been deposited to Figshare (https://doi.org/10.6084/m9.figshare.7243463.v1).

## APPENDIX 1

Average body weight and total length of 135 individuals of Chinese giant salamander. Multiple comparisons of different ages were analysed by Kruskal–Wallis test; a, b, c mean group differences

**Table:**

Age & Number	Body weight (g)	Total length (cm)
1, *n* = 32	0.5 ± 0.1^a^	4.0 ± 0.4^a^
2, *n* = 89	7.0 ± 1.0^b^	10.8 ± 1.0^a^
3, *n* = 7	860.0 ± 120.0^c^	51.9 ± 3.3^b^
4, *n* = 7	1501.0 ± 136.0^c^	64.3 ± 3.0^c^

## APPENDIX 2

The sample information

**Table:**

#SampleID	Location	age	Pooling information (individual)
D.2.1	Duodenum2	HHY2	ID10–ID103
D.3.5	Duodenum3	HHY3	ID6
D.3.4	Duodenum3	HHY3	ID7
D.3.12	Duodenum3	HHY3	ID8
D.3.6	Duodenum3	HHY3	ID9
D.4.1	Duodenum4	HHY4	ID1
D.4.2	Duodenum4	HHY4	ID2
D.4.16	Duodenum4	HHY4	ID3
D.4.22	Duodenum4	HHY4	ID4
D.4.23	Duodenum4	HHY4	ID5
HHY.1	HHY1	HHY1	ID104–135 (overall digestive tract)
I.2.1	Ileum2	HHY2	ID10–ID28
I.2.2	Ileum2	HHY2	ID29–ID54
I.2.3	Ileum2	HHY2	ID55–ID79
I.2.4	Ileum2	HHY2	ID80–ID103
I.3.5	Ileum3	HHY3	ID6
I.3.4	Ileum3	HHY3	ID7
I.3.12	Ileum3	HHY3	ID8
I.3.6	Ileum3	HHY3	ID9
I.4.1	Ileum4	HHY4	ID1
I.4.2	Ileum4	HHY4	ID2
I.4.16	Ileum4	HHY4	ID3
I.4.22	Ileum4	HHY4	ID4
I.4.23	Ileum4	HHY4	ID5
R.2.1	Retcum2	HHY2	ID10–31
R.2.2	Retcum2	HHY2	ID32–53
R.2.3	Retcum2	HHY2	ID54–69
R.2.4	Retcum2	HHY2	ID70–87
R.2.5	Retcum2	HHY2	ID88–ID103
R.3.5	Retcum3	HHY3	ID6
R.3.4	Retcum3	HHY3	ID7
R.3.12	Retcum3	HHY3	ID8
R.3.6	Retcum3	HHY3	ID9
R.4.1	Retcum4	HHY4	ID1
R.4.2	Retcum4	HHY4	ID2
R.4.16	Retcum4	HHY4	ID3
R.4.22	Retcum4	HHY4	ID4
R.4.23	Retcum4	HHY4	ID5
S.2.1	Stomach2	HHY2	ID10–22
S.2.2	Stomach2	HHY2	ID23–39
S.2.3	Stomach2	HHY2	ID40–54
S.2.4	Stomach2	HHY2	ID55–68
S.2.5	Stomach2	HHY2	ID69–87
S.2.6	Stomach2	HHY2	ID88–ID103
S.3.5	Stomach3	HHY3	ID6
S.3.4	Stomach3	HHY3	ID7
S.3.12	Stomach3	HHY3	ID8
S.3.6	Stomach3	HHY3	ID9
S.4.1	Stomach4	HHY4	ID1
S.4.2	Stomach4	HHY4	ID2
S.4.16	Stomach4	HHY4	ID3
S.4.22	Stomach4	HHY4	ID4
S.4.23	Stomach4	HHY4	ID5

## APPENDIX 3

Rarefaction curve for these 53 gastrointestinal samples based on QIIME 2 (DADA2)

## APPENDIX 4

Number of bacterial taxa observed at the phylum, family and genus level

**Table:**

Age	Phylum	Family	Genus
1	20	135	280
2	23 ± 7	161 ± 59	286 ± 84
3	29 ± 7	196 ± 53	372 ± 100
4	30 ± 6	217 ± 76	422 ± 143

## APPENDIX 5

Bray‐Curtis distance‐based UPGMA clustering of 53 samples in this study

## APPENDIX 6

The comparisons of UniFrac‐unweighted distances between microbiota of four gut chambers (stomach, duodenum, ileum and rectum) (within or between groups)

## APPENDIX 7

The one‐way PERMANOVA test for either location or age of samples in this study using Bray‐Curtis distance (Bonferroni‐corrected p values)

**Table:**

Location		Bonferroni‐corrected *p* value				
PERMANOVA		Location	Duodenum	Ileum	Retcum	Stomach
Permutation *N*:	9,999	Duodenum		0.063	**0.0006**	1
Total sum of squares:	4.822	Ileum	0.063		1	0.1428
Within‐group sum of squares:	4.061	Rectum	**0.0006**	1		**0.0012**
*F*:	2.998	Stomach	1	0.1428	**0.0012**	
*p* (same):	0.0003					
**Age**		**Bonferroni‐corrected *p* value**				
**PERMANOVA**		**Age**	**HHY2**	**HHY3**	**HHY4**	
Permutation *N*:	9,999	HHY2		**0.0003**	**0.0003**	
Total sum of squares:	4.822	HHY3	**0.0003**		0.9963	
Within‐group sum of squares:	4.002	HHY4	**0.0003**	0.9963		
*F*:	5.023					
*p* (same):	0.0003					

## APPENDIX 8

The phylum level of these 53 samples in this study. HHY1: age 1 year. HHY2: age 2 years. HHY3: age 3 years. HHY4: age 4 years

## APPENDIX 9

Relative abundance of the dominant bacterial phyla which were significantly dissimilar from age 2 to 4; labelled as HHY2, HHY3, and HHY4

## APPENDIX 10

Relative abundance of top 10 bacterial taxa that were observed at the level of genus from age 2 to 4 years

**Table:**

Taxa	Age 2		Age 3		Age 4
	Relative abundance (%)	Taxa	Relative abundance (%)	Taxa	Relative abundance (%)
Cetobacterium	2.75	Mycoplasma	3.12	Mycoplasma	2.35
Bacteroides	1.42	Halomonas	1.61	Ruminococcaceae NK4A214 group	1.38
Aeromonas	0.94	Ruminococcaceae NK4A214 group	1.28	Halomonas	1.29
Clostridium sensu stricto 1	0.79	Citrobacter	0.56	Candidatus Amphibiichlamydia	0.72
Citrobacter	0.72	Lactobacillus	0.52	Lactobacillus	0.601
Paraclostridium	0.38	Cetobacterium	0.47	Thauera	0.45
Muribaculaceae_norank	0.36	Shewanella	0.46	Muribaculaceae_norank	0.43
Acinetobacter	0.35	Unclassified	0.42	Shewanella	0.37
Clostridium sensu stricto 5	0.33	Candidatus Amphibiichlamydia	0.30	Flavobacterium	0.37
Parabacteroides	0.31	Bacteroides	0.29	Bacteroides	0.36

## APPENDIX 11

Relative abundance of the dominant bacterial phyla at the same age (3 and 4) which were significantly dissimilar among D: duodenum; I: ileum; R: rectum; S: stomach
